# Unicompartmental Knee Arthroplasty: Minimal Important Difference and Patient Acceptable Symptom State for the Forgotten Joint Score

**DOI:** 10.3390/medicina57040324

**Published:** 2021-04-01

**Authors:** Umile Giuseppe Longo, Sergio De Salvatore, Vincenzo Candela, Alessandra Berton, Carlo Casciaro, Gaia Sciotti, Giada Cirimele, Anna Marchetti, Ilaria Piergentili, Maria Grazia De Marinis, Vincenzo Denaro

**Affiliations:** 1 Department of Orthopaedic and Trauma Surgery, Campus Bio-Medico University, Via Alvaro del Portillo, 200, Trigoria, 00128 Rome, Italy; s.desalvatore@unicampus.it (S.D.S.); v.candela@unicampus.it (V.C.); a.berton@unicampus.it (A.B.); c.casciaro@unicampus.it (C.C.); ilaria.piergentili94@gmail.com (I.P.); denaro@unicampus.it (V.D.); 2 Research Unit Nursing Science, Campus Bio-Medico di Roma University, 00128 Rome, Italy; sciotti.cbm@gmail.com (G.S.); giacirimele@gmail.com (G.C.); a.marchetti@unicampus.it (A.M.); m.demarinis@unicampus.it (M.G.D.M.)

**Keywords:** MCID, PASS, Forgotten Joint Score-12, FJS-12, unicompartmental knee arthroplasty, UKA

## Abstract

*Background and Objectives*: Unicompartmental knee arthroplasty (UKA) is a valid alternative to total knee arthroplasties (TKAs) in selected cases. After surgery, patients’ experience and satisfaction were traditionally evaluated by pre- and postsurgical scores and Patient-Reported Outcome Measures (PROMs). Otherwise, a statistically significant change does not necessarily correlate to a clinically meaningful improvement when measured using PROMs. To evaluate the real effect of a specific treatment and understand the difference between groups in a clinical trial, it is necessary to use a meaningful quantum of change on the score assessed. The minimal clinically important difference (MCID) and the Patient Acceptable Symptom State (PASS) can provide this meaningful change. This paper aimed to calculate the MCID and the PASS of the Forgotten Joint Score (FJS-12) after UKA. *Materials and Methods:* A total of 40 patients with a mean age 72.5 ± 6.4 years undergoing UKA were assessed preoperatively and six months postsurgery using the FJS-12 and the Oxford Knee Score (OKS). The baseline and 6-month postoperative scores were compared using the Wilcoxon signed ranks test. The correlation was calculated with Spearman’s rho. Both distribution-based approaches and anchor approaches were used to estimate MCID for the FJS-12. The 75th percentile and the Receiver operating characteristic (ROC) curve methods were used to calculate the PASS of FJS-12. *Results:* MCID estimates for normalized FJS-12 for UKA ranged from 5.68 to 19.82. The threshold of the FJS-12 with ROC method was 72.92 (AUC = 0.76). The cut-off value computed with the 75th percentile approach was 92.71. *Conclusions*: The MCID and PASS represent valid tools to assess the real perception of clinical improvement in patients who underwent UKA. The MCID value of FJS-12 was 12.5 for patients who underwent UKA. The value of the PASS for the FJS-12 in patients who underwent UKA was 72.92.

## 1. Introduction

Unicompartmental knee arthroplasty (UKA) is a valid alternative to total knee arthroplasties (TKAs) in selected cases [1,2]. In patients with similar surgical indications, UKA represents numerous advantages compared to TKA, such as faster recovery, reduced blood loss and pain [3,4]. After surgery, patients’ experience and satisfaction were traditionally evaluated by pre- and postsurgical scores [5,6], implant survival, radiographic assessment, clinical examination and Patient-Reported Outcome Measures (PROMs). PROMs usually consist of a validated questionnaire that assesses the patient perception of a specific treatment of disease [7]. The efficacy of a surgical procedure or a specific treatment is usually evaluated as the improvement in some predetermined outcome measure [8]. Otherwise, a statistically significant change does not necessarily correlate to a clinically meaningful improvement when measured using PROMs. They could be influenced by a patient’s mood, feelings and perspectives [7]. PROMs are useful as they provide a direct input about patients’ perceptions that are not possible to obtain by other methods. The Oxford Knee Score (OKS) and the Western Ontario and McMaster University Osteoarthritis Index (WOMAC) are two of the most commonly adopted questionnaires to assess the PROMs after TKA or UKA [9,10,11,12]. However, patients treated by UKA usually reported better functional outcomes compared to TKA [13]. This factor could lead to a high ceiling effect if assessed by traditional PROMs (WOMAC and OKS). In other words, many patients could report the highest possible score on the PROM scale [5]. The Forgotten Joint Score (FJS-12) is a valid and responsive PROMs scale created to evaluate prosthesis awareness during daily activities [14,15]. Developed by Behrend et al. in 2012, it assesses the degree of awareness of the prosthetic joint with a low ceiling effect [15,16]. Therefore, this score could more accurately stratify patients who achieve superior results with traditional PROMs [15]. Moreover, the FJS-12 has been translated into and validated in many languages and is widely used in clinical practice and research [17,18]. 

PROMs are usually numerical data (generally range between 0 and 100) that are difficult to interpret. To evaluate the real effect of a specific treatment and understand the difference between groups in a clinical trial, it is necessary to use a meaningful quantum of change on the score assessed. Moreover, this score should be contextualized to establish if statistically significant results could be identified as clinically relevant. 

The minimal clinically important difference (MCID) for a PROM can provide this meaningful change [19]. 

The minimal clinically important difference (MCID) was first described by Jaeschke et al. in 1989 [20]. They defined the MCID as “the smallest difference in score in the domain of interest which patients perceive as beneficial and which would mandate, in the absence of troublesome side effects and high cost, a change in the patient’s management”. Similar concepts to the MCID are the Minimum Important Change (MIC) and the minimal important difference (MID) [14,21,22]. These parameters are always used interchangeably with MCID, causing ambiguity [23]. The MCID has been used in clinical trials as a benchmark to assess the amount of PROMs scale improvement, reflecting clinical significance. Nowadays, PROMs are increasingly used in clinical studies; therefore, MCID represents a useful and valid tool in setting numerical thresholds for significant clinical improvement [8]. Both distribution-based and anchor methods could be used to assess the MCID [6].

The Patient Acceptable Symptom State (PASS) is considered as the minimum PROM cut-off value that corresponds to a patient’s satisfactory state of health. The PASS is the threshold on a PROM most closely associated with patient satisfaction, assessed by a separate questionnaire (the anchor) [23]. It is an important value because the surgeon can interpret it as a clinically relevant treatment target [5]. 

Despite that the validity and reliability of the FJS-12 has already been demonstrated [14], the MCID of this score remains unknown for UKA. Moreover, only one study assessed the PASS of FJS-12 for UKA [5]. Therefore, this paper aimed to calculate the MCID and the PASS of FJS-12 after UKA. Distribution-based approaches and anchor approaches, using OKS as anchor, were used.

## 2. Materials and Methods

This is a quality improvement study. Patients undergoing UKA from January 2019 to October 2019 were assessed preoperatively and six months postsurgery using the FJS-12 and the OKS. Preoperative outcome questionnaires and follow-up questionnaires at six months postoperation were completed by 40 patients (26 females and 14 males), with a mean age of 72.5 ± 6.4 years and mean Body Mass Index (BMI) of 27.8 ± 4.34. All the patients included reported radiographic and clinical findings of unicompartmental knee osteoarthritis (grades 3–4 according to Kellgren Lawrance Classification) [24] and were treated by the same senior surgeon. All the procedures performed were unicompartmental knee arthroplasty with fixed bearing. Only primary implants were considered. All the procedures were performed in a public health setting. Exclusion criteria revisions were the presence of symptoms or signs of inflammatory arthritis [25]. Nowadays, anterior cruciate ligament injury, anteroposterior instability, BMI > 30 and flexion contracture are defined as minor contraindication for UKA [25], therefore they were not considered as major contraindications. 

The study was conducted according to the guidelines of the Declaration of Helsinki [26], and approved by the Institutional Review Board of Campus Bio-Medico University of Rome (COSMO study, Protocol number: 78/18 OSS ComEt CBM, 16/10/18).

### 2.1. Assessment Instruments

#### 2.1.1. FJS-12

The Forgotten Joint Score-12 is a PROM scale designed to assess prosthesis awareness during daily activities (i.e., the patient’s ability to forget the presence of a prosthesis) [14]. This consists of 12 questions, with a five-point Likert response format, which are summed to obtain scores ranging from 12 to 60. The raw score was normalized to a range from a minimum of 0 to a maximum of 100 points. High scores indicate a good outcome.

#### 2.1.2. OKS

The Oxford Knee Score is a clinical orthopaedic score used to assess the patient’s valuation of their knee-related health status after the surgery [27]. It is composed of 12 questions, with a zero (worse outcome) to four (best outcome) Likert scale response, which is summed to obtain scores ranging from 0 (worse condition) to 48 (best condition). 

### 2.2. Statistical Analysis

A priori power analysis was performed for sample size estimation. A sample size of 15 patients provides 80% power (alpha = 0.05, two-sided) to detect a difference with a large effect size (Cohen’s d = 0.8) in FJS-12 in the preoperative period to the 6-month postoperative follow-up, as in the literature [14,28]. Data normality for the FJS-12 was assessed using the Shapiro–Wilk test of normality. The non-normal distributions of the FJS-12, the baseline and 6-month postoperative scores were compared using the Wilcoxon signed ranks test; the correlation was calculated with Spearman’s rho. Values of *p* < 0.05 were considered statistically significant. Both distribution-based approaches and anchor approaches were used to estimate MCID for the FJS-12. Statistical analyses were performed using SPSS version 26 (IBM Inc., Armonk, NY, USA).

#### 2.2.1. Distribution-Based Approach for MCID

Different approaches were calculated: 0.5 standard deviations (0.5 SDs) were used for a medium effect size; the standard error of measurement (SEM), the smallest change above the measurement error and the minimum detectable change (MDC), and the smallest change above the measurement error had 95% confidence intervals. The SEM formula was SEM = SD×√(1−r) where r is Cronbach’s α, a measure of reliability. The MDC formula was MDC = SEM×1.96×√2

#### 2.2.2. Anchor Approaches for MCID

At the 6-month follow-up, each patient answered the following surgical question “How do you feel after the surgical intervention carried out?”. This question was used as anchor to determine a clinically meaningful change in the outcome. Responses were based on the 5-point global scale that ranged from –2 (“much worse”) to +2 (“much better”). Patients who responded “much worse” (−2), “a little worse” (−1) or “equal” (0) represented the “No change group”. Those responding with “a little better” (+1) represented the “Minimally improved group”. The MCID cut-offs were calculated by comparing the “No change group” with the “Minimally improved group”. As a secondary check for the determination of the MCID with the anchor approach, analogous to Den Oudsten et al. [29] and Kwakkenbos et al. [30], the change in a PROMS was used. We chose the change in OKS. Since Beard et al. found that the MCID of OKS was 5 [31], the “Minimally improved group” and “No change group” were categorized as patients with an increase of at least 5 in OKS and patients with a change score < 5, respectively. An external anchor was defined as valid if the correlation coefficient with FJS-12 was at least 0.3 with *p* < 0.05 [32]. Three anchor methods were used: receiver operating characteristic (ROC) curves, the Change Difference (CD) and the Mean Chance (MC). The ROC curves were used to identify the change in FJS-12 that maximized sensitivity and specificity (with Youden Index). With the ROC method, the Area Under the Curve (AUC) was also calculated, which is a measure of the ability of a questionnaire to distinguish patients who have and have not changed, according to external criteria. An AUC of 1.0 describes a test with perfect discriminatory ability (100% sensitivity and 100% specificity). An AUC of 0.50 describes a test with no discriminatory value (50% sensitivity and 50% specificity). Terwee et al. recommended a criterion value of 0.70 or more to define a valid anchor [33]. The CD in FJS-12 was the mean difference between patients with change in OKS higher than 5 and patients with change in OKS less than 5 between the preoperative period and 6-month postoperative follow-up scores. The MC in FJS-12 is the mean difference in patients who reported change in OKS score higher than 5.

#### 2.2.3. Calculation Methods for PASS

The anchor question for PASS should relate pain, physical function and satisfaction of patients, as suggested in the literature [22]. “In general, would you say that your health is at least good?” was the anchor question for calculating the PASS values. The possible responses were “Yes” or “No”. Patients who responded “Yes” were considered as satisfied with their symptom state. PASS thresholds of FJS-12 were calculated using the 75th percentile of the cumulative percentage curve of patients who consider themselves as having an acceptable state of symptoms and the point on the ROC curve where the cut-off was calculated using the Youden index. 

## 3. Results

The Shapiro–Wilk test showed non-normal FJS-12 distribution at baseline and 6 months postoperation (*p* < 0.05).

The average FJS-12 score improved from 69.1 ± 15 at baseline to 83.3 ± 11.4 at 6-month postoperative follow-up. The values of FJS-12 at baseline ranged from a minimum of 25 to a maximum of 89.6, with a 0% floor and ceiling effects. At the 6-month follow up, the FJS-12 score ranged from a minimum of 56.3 to a maximum of 100, with a 0% floor effect and 5% ceiling effect. A statistically significant change between preoperative and 6-month follow-up scores was found (*p* < 0.001). 

A high value of Cronbach’s alpha (α = 0.9) was calculated. Since the correlation between FJS-12 change and OKS change was higher 0.3 (r = 0.3, *p* = 0.044), change in OKS was defined as valid external anchor to calculate MCID. The AUC calculated with the surgical question (“How do you feel after the surgical intervention carried out?”) was 0.2, therefore this is not an adequate anchor.

MCID estimates for normalized FJS-12 for UKA ranged from 5.7 to 19.8. MCID values, calculated with a distribution-based approach, were 8.8 with 0.5 SD, 5.7 with SEM and 15.7 with MDC. The MCID values calculated with anchor approaches were 12.5 (AUC = 0.8) with the ROC method, 19.8 with the CD method and 15.7 with the MC method. The AUC calculated with change in OKS anchor was higher than 0.7, therefore change in OKS was an adequate anchor.

An MCID of 8.8 was a change with a medium effect size (with effect size = 0.5). An MCID of 5.7 was a change with a high internal consistency (α = 0.9), and 15.7 was the smallest change above the measurement error with a confidence interval of 95%. A value of 12.5 was the MCID with maximized sensitivity and specificity, with a high instrument of responsiveness (AUC = 0.8). A value of 19.8 was the mean difference of FJS-12 between preoperative and 6-month postoperative results in responders. Lastly, 15.7 was the mean difference between patients in the “Minimally improved group” and patients in the “No change group”. All the results are reported in Table 1.

**Figure 1:**
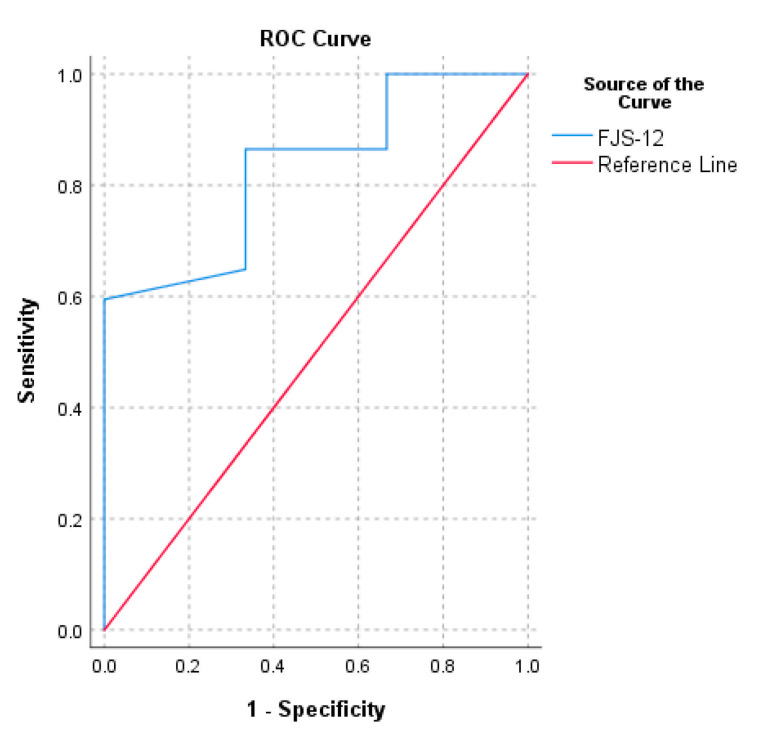
Receiver operating characteristic curve (ROC) for the prediction of Forgotten Joint Score (FJS-12)’s MCID based on the change in Oxford Knee Score (OKS) (> 5).

**Figure 2:**
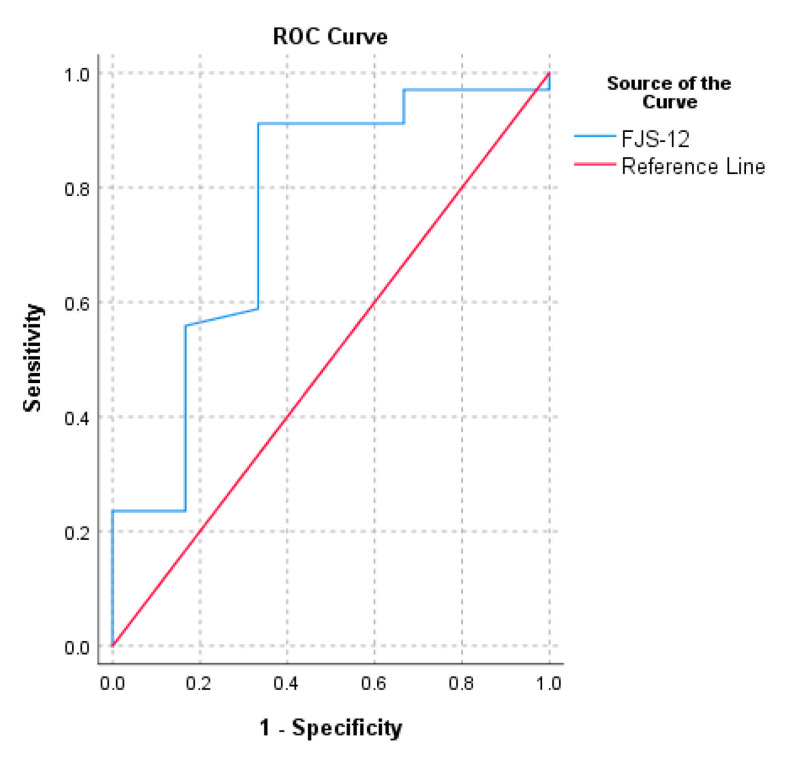
Receiver operating characteristic curve (ROC) for the prediction of FJS-12′s PASS based on the anchor question “In general, would you say that your health is at least good?”.

The PASS calculated for normalized FJS-12 for UKA ranged from 72.9 to 92.7. The threshold of the FJS-12 that maximized the sensitivity and specificity for detecting a PASS was 72.9 (AUC = 0.8). The cut-off value computed with the 75th percentile approach was 92.7 (Table 2). The ROC curve of FJS-12′s MCID based on the change in OKS and the ROC curve of FJS-12′s PASS are reported in Figure 1 and Figure 2, respectively.

## 4. Discussion

In the present study, data from 40 people who underwent UKA were analyzed. The aim of this study was to find the FJS-12′s MCID and PASS from the preoperative period to the 6-month postoperative follow-up in patients who received a UKA.

The MCID is a useful tool to assess the smallest change that a patient could perceive as beneficial, and it should be essential for clinicians to have to decide on the therapeutic process [22]. 

Ingelsrud et al. reported an MCID of the FJS-12 in TKA between 14 and 23 points [27]. Robinson et al. assessed the MCID of 15 points for FJS-12 in total hip arthroplasty [34]. Behrend et al. calculated an FJS-12′s MCID of 13 in anterior cruciate ligament reconstruction [35]. For patients who underwent UKA, only MCIDs of OKS and SF-36 were found [36,37,38]. 

Different methods to compute MCID were reported and were divided into distribution-based approaches and anchor approaches. The distribution-based approaches are built upon the statistical properties of a study’s result [39] and include the 0.5 SD method, the SEM and the MDC. The 0.5 SD represents a clinically meaningful change with a medium effect size [40]. The SEM is the change due to unreliability of the scale or measurement errors [41]. A difference smaller than the calculated SEM is probably due to a measurement error rather than a real observed change; therefore, an MCID value smaller than the SEM likely results from error. The MDC is the smallest change that can be considered above the measurement error and has a 95% level of confidence [42]. An MCID value smaller than the MDC was not considered valid. The MCID was determined using the change in OKS (>5) as the anchor. 

Instead, the most frequently used anchor approaches are the ROC, the CD and the MC methods. The first identifies the MCID as the point of the receiver operating characteristics curve in which sensitivity and specificity are maximized. This cut-off was found with Youden’s Index [43]. The second assessed the difference between the change in the average score of improved and nonimproved patients. The last calculated the mean difference between the two time points in patients with minimal improve.

No agreement has been achieved as to which MCID calculation method is superior. To account for the method and sample-dependent variations, identifying a single threshold that defines the MCID is potentially misleading. Therefore, it is recommended that a plausible range of MCIDs be presented [44]. Consequently, both distribution-based approaches and anchor approaches were used to assess the MCID of FJS-12.

The MCID ranged from 5.7 to 19.8 points in patients who underwent UKA. According to Copay et al., a valid MCID should at least be larger than the SEM and correspond to the patient perception of the importance of the change [45]. Based on these criteria, the ROC, CD and MC approaches appears to be the more appropriate calculation methods for defining an MCID threshold. The 0.5 SD and MDC methods were also higher than the SEM, but anchoring approaches are most commonly used in orthopedic studies [46,47,48]. However, according to Maredupaka et al. [48] the ROC method was the most suitable for “within an individual” analysis in clinical practice. Based on these premises, the MCID value of FJS-12 was 12.5 for patients who underwent UKA.

The second purpose of the present study was to evaluate the PASS value of FJS-12 at 6 months. The PASS is the cut-off on a PROM most closely associated with patient satisfaction, which is assessed by a separate questionnaire [23]. The most used approaches to calculate the PASS are the 75th percentage approach and the receiver operating characteristic (ROC) curves [22]. The estimated values of PASS ranged from 72.9 to 92.7. Wang et al. reported a PASS value of 40.6 for FJS-12 after UKA [5], while a score above 84.4 can be interpreted as having achieved a forgotten joint [5]. Usually, the mean follow-up of a study is 21.5 months, but in our study it was 6 months. Within the results calculated, the most valid was the one assessed using the ROC method. The value of the PASS for the FJS-12 in patients who underwent UKA was 72.9.

This study has several strengths. Firstly, this is the first study on the MCID of FJS-12 in patients who underwent UKA. Secondly, the score used as anchor is valid and commonly adopted in the literature. Finally, the most common ad hoc methods were used to calculate MCID and PASS. However, this paper also has limitations. Firstly, MCID and PASS were calculated for a 6-month follow-up. Further studies with longer follow-ups are required. Secondly, since only one anchor was accessible for use in the present study, the consistency of the results across different anchors was not assessed. Lastly, even if the sample size was sufficient according to the power analysis, a higher number of patients has been used in the literature.

## 5. Conclusions

The FJS-12 score represents a valid and sensible PROMs score with a low ceiling effect. The low ceiling effect solves the limits of other scores such as WOMAC and OKS when detecting small clinical changes in patients who report good results. The MCID and PASS represent valid tools to assess the real perception of clinical improvement in patients who underwent UKA. These tools are very useful nowadays, being more representative of what is really clinically significant for a patient, instead of a simple statistical significance improvement between pre- and postoperative scores. The MCID value of FJS-12 was 12.5 for patients who underwent UKA. The PASS value of FJS-12 in patients who underwent UKA was 72.9.

## Figures and Tables

**Table 1:** Summary of the results of minimal clinically important difference (MCID).

MCID	Cut-Off Value	Anchor
0.5 SD	8.8	/
SEM	5.7	/
MDC	15.7	/
ROC (AUC)	12.5 (0.8)	OKS > 5
CD	19.8	OKS > 5
MC	15.7	OKS > 5

0.5 SD: 0.5 standard deviation; CD: Change Difference; MC: Mean Change; MCID: minimum clinically important difference; MDC: minimum detectable change; ROC/AUC: Receiver operating characteristic; SEM: Standard Error of Measurement. OKS: Oxford Knee Score

**Table 2:** Summary of the results of the Patient Acceptable Symptom State (PASS).

PASS	Cut-Off Value	Anchor
ROC (AUC)	72.9 (0.8)	In general, would you say that your health is at least good?
75th percentile	92.7	In general, would you say that your health is at least good?

PASS: Patient Acceptable Symptom State; ROC/AUC: Receiver operating characteristic.

## Data Availability

The data presented in this study are available on request from the corresponding author. The data are not publicly available due to privacy.

## Abbreviations

0.5 SD	0.5 standard deviation
CD	Change Difference
FJS-12	Forgotten Joint Score-12
MC	Mean Change
MCID	Minimum Clinically Important Difference
MDC	minimum detectable change
MIC	Minimum Important Change
OKS	The Oxford Knee Score
PASS	Patient Acceptable Symptom State
PROMs	Patient-reported outcome measure
ROC/AUC	Receiver operating characteristic
SEM	Standard Error of Measurement
SF-36	Short Form Health Survey
TKA	Total Knee Arthroplasty
UKA	Unicompartmental Knee Arthroplasty
WOMAC	Western Ontario and McMaster University Osteoarthritis Index
